# *Mentha longifolia* syrup in secondary amenorrhea: a double-blind, placebo-controlled, randomized trials

**DOI:** 10.1186/2008-2231-20-97

**Published:** 2012-12-21

**Authors:** Roshanak Mokaberinejad, Nafiseh Zafarghandi, Soodabeh Bioos, Fataneh Hashem Dabaghian, Mohsen Naseri, Mohammad Kamalinejad, Gholamreza Amin, Ali Ghobadi, Mojgan Tansaz, Ali Akhbari, Mohammadali Hamiditabar

**Affiliations:** 1 Department of Traditional Medicine, Faculty of Medical Science, Shahed University, Tehran, Iran; 2 Department of Gynecology and Obstetrics, Faculty of Medical Science, Shahed University, Abdallahzade Street, Tehran, Iran; 3 Faculty of Traditional Medicine, Tehran University of Medical Sciences, Tehran, Iran; 4 Research Institute for Islamic & Complementary Medicine, Tehran University of Medical Sciences, Tehran, Iran; 5 Traditional Medicine Clinical Trial Research center, Shahed University, Tehran, Iran; 6 Department of Pharmacognosy, School of Pharmacy Shaheed Beheshti University of Medical Sciences, Tehran, Iran; 7 Department of traditional pharmacy, Faculty of traditional medicine, Tehran University of Medical Sciences, Tehran, Iran; 8 School of Traditional Medicine, Shaheed Beheshti University of Medical Sciences, Tehran, Iran; 9 Ashrafi Esfahani Hospital, Shaheed Beheshti University of Medical Sciences, Tehran, Iran; 10 Excel Diagnostics and Nuclear Oncology Centre, Houston, Texas

**Keywords:** Mentha longifolia, Lamiacea, Amenorrhea, Oligomenorrhea, Iranian traditional medicine, Ehtebas tams

## Abstract

**Background:**

Amenorrhea is defined as the cessation of menses. Hormone therapy is the most common treatment. Due to the contraindications and side effects of it and the increasing demand for alternative medicine substitutes, *Mentha longifolia L.* was used in this study. *Mentha longifolia L.* is a known medication in Iranian traditional medicine to induce menstrual bleeding in women with secondary amenorrhea and oligomenorrhea.

**Methods:**

A double-blind, randomized, placebo-controlled, multicenter study was conducted in 120 women with secondary amenorrhea and oligomenorrhea. Treatment consisted of sequential oral syrup, 45 ml (15 ml three times a day) for 2 weeks. If the patients did not have menstruation after 2 weeks of taking the medication, we would wait for two more weeks. If the patients had menstruation at each stage of using the drug, we started it one week after the end of menstruation. But if the patients had not menstruate after four weeks (two-week using of drug and waiting for two more weeks), the previous steps were repeated. The drug and placebo were repeated in three cycles of menstruation. Bleeding was documented by the patient on diary cards. The primary outcome variable was the occurrence (yes/no) of bleeding during the first treatment cycle. The secondary efficacy outcome was the regularity of bleeding pattern during the three cycles of the study.

**Results:**

The number of women with bleeding during the first cycle were higher in the drug group as in the placebo group (68.3% vs. 13.6%; p < 0.001). The regularity of bleeding throughout the study was markedly better in the drug group compared with those given placebo (33.3% vs. 3.3%; p < 0.001). No notable complication or side effect was reported in relation to *Mentha longifolia L.* syrup.

**Conclusion:**

In conclusion, *Mentha longifolia L.* syrup is a safe, well-tolerated, and effective choice in inducing bleeding and maintaining regular bleeding in women with secondary amenorrhea and oligomenorrhea.

## Introduction

Secondary amenorrhea (SA) is defined as the cessation of menses for 6 months in female previously irregular menstrual pattern, or the cessation of menses for 3 consecutive months [1]. It has, on the other hand, been specifically defined in various ways, some of which overlap with oligomenorrhea (infrequent menstrual flow at intervals of 39 days to 6 months or 5–7 cycles in a year) [2,3]. The overall prevalence of secondary amenorrhea in among women of reproductive age is around 3% [4] and prevalence of oligomenorrhea is 10.2% [5].

The most common form of secondary amenorrhea is hypogonadotrophic disorders caused by hypothalamic suppression, particularly functional hypothalamic amenorrhea (FHA), due to various stressors with no evidence of systemic/endocrine causal factors [6,7]. Another common form of secondary amenorrhea is ovulatory disorders often associated with polycystic ovary syndrome (PCOS) [3,8]. PCOS is the most common endocrine disorder in women [5,9].

Treatment of secondary amenorrhea and oligomenorrhea is mostly based on empiric hormone with estrogen and progesterone [10]. Several complications have been reported by hormone therapy [11].

One of the most influential Iranian physician between 9th and 14th centuries AD was Ibn –Sina or Avicenna (980 – 1037 A.D). His chief medical book is “Al-Qanon fi Al-Tibb” or “Canon of Medicine” [12,13]. The twenty-first chapter of third book of “Canon of Medicine” deals principally with various kinds of uterine diseases [14]. In this section, amenorrhea and oligomenorrhea is described under a same title: “Ehtebas Tams” [15]. “Ehtebas tams” is defined as the absence of menstruation [14,16].

Based on Iranian traditional medicine (ITM) texts particularly “Canon of medicine” and “Al-Havi” (Rhazes 865–925 A.D), *Mentha longifolia* is one of the medicinal herbs that can influence menstrual periods [14,17].

*Mentha longifolia* (L.) a member of the Lamiacea (Labiatae) family, whose habitat is from Southeast Asia and is known as Horse mint or wild mint [18]. The historical use of it is not different from its use in modern herbal medicine. It has been reported as a remedy for common cold, cough, sinusitis, fever, bronchitis, indigestion, intestinal colic, loss of appetite, nausea and vomiting also it has other various uses in ITM that are unknown in modern medicine like its indication for menstrual disorders. *Mentha longifolia* known as Fudanaj or Pooneh in ITM is a common constituent of the Middle East diet [19]. However, its effect on menstruation has not been scientifically evaluated yet.

The aim of the present study is to compare the efficacy of sequential *Mentha longifolia* syrup with that of placebo in inducing regular bleeding in women with secondary amenorrhea or oligomenorrhea and normal FSH, TSH, Prolactin levels.

This is the first double-blind, placebo-controlled, randomized study to assess *Mentha longifolia* syrup induced bleeding in this specific diseased condition.

## Materials and methods

### Patients

This multicenter double-blind, randomized study (6 centers in Tehran and Qom, Iran) was carried out among 120 women with secondary amenorrhea or oligomenorrhea (cessation of bleeding for at least 60 days without pregnancy).

The patients were between 18 and 35 years old and had premenopausal levels of follicle-stimulating hormone (FSH) (<20 IU/l). Patients with abnormal prolactine, abnormal thyroid function tests, and congenital adrenal hyperplasia were excluded. Other exclusion criteria included clinically significant diseases that might have limited participation in or completion of the study including any anatomical abnormality or gynaecological neoplasia, a positive pregnancy test, breastfeeding, severe drug allergy or history of severe unusual drug reactions towards herbs and intake of any hormonal products (chemical or herbal) in the previous 2 months.

All patients provided written informed consent. The Ethics Committee of Shahed University approved the protocol (approval number: 4/112251). Further, the trial was registered in the Iranian Registry of Clinical Trials with the number IRCT201110027690 N1.

### Preparations of *Mentha longifolia L*

*Mentha longifolia L.* dried leaves were purchased from local market in Tehran bazar, the center of Tehran province, Iran and identified by professor Gholamreza Amin, and kept at the herbarium of faculty of pharmacy, Tehran University of Medicinal Sciences, under the voucher number PMP - 308.

### Volatile analysis of *Mentha longifolia L*

The dried plant were used for extraction of total essential oil using Clevenger apparatus and yielded 1 ml/100 mg of dried plant. Analysis of total essential oil showed some major component as; 1, 8 cineole (11.58%), pulegone (21.90%), piperitone oxide (42.51%) and caryophyllene oxide (3.64%).

### Preparations of syrup and placebo

Total evaporated etanolic extract of *Mentha longifolia L.* used for preparation of syrup under supervision of professor Kamalinejad at traditional pharmacy department of faculty of traditional medicine, Tehran University of Medicinal Sciences, Tehran- Iran while each 5 ml of syrup contains 300 mg ethanol extract of plant dried powder in the base of sucrose pharmacopoeal syrup.

The placebo was prepared in the same appearance, with the base of sucrose pharmacopoeal syrup without *Mentha longifolia L.* extract.

### Biochemical examinations

Blood samples were taken from all patients for evaluation of the hormonal status including total testosterone, free testosterone and FSH, LH, TSH and prolactin serum levels. The blood samples (10 mL) were collected from each of the patients after a 12 h overnight fasting. The serum was separated by centrifugation at 3000 rpm for 10 min after keeping at room temperature for 15 min and were measured by using the Roch diagnostics® chemiluminescence Immunoassay (CIA) analyzer.

### Treatment and assessments

The patients were randomized in two groups (Drug or placebo) to receive sequential oral syrup 45 ml three times a day for 2 weeks. In the event of no menstruation in two weeks, the patient was given the medication for two more weeks. After four weeks of having no menstruation, the patient was instructed to take her medication for extra two weeks and repeated the previous steps.

In the event of bleeding in each stage of therapy, the patient continued taking the medication one week after the end of menstruation and restart it again.

**Figure 1:**
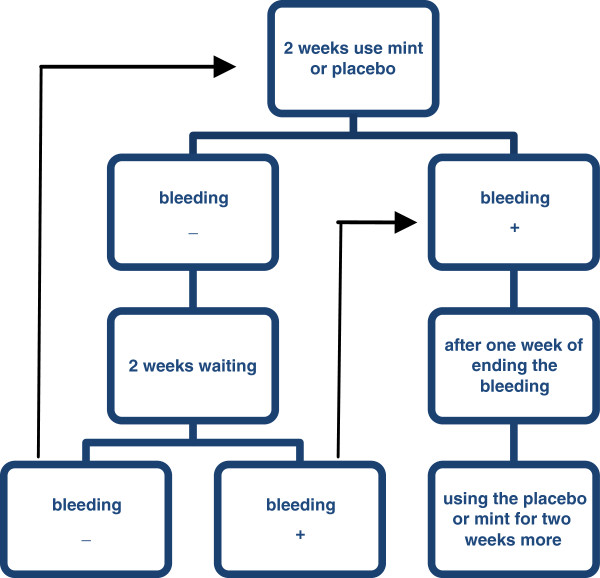
Methods of use of mint or placebo in the study.

The drug and placebo intake were repeated in three cycles of menstruation (Figure 1). Then the patients had to report any signs of bleeding immediately.

Randomization was affected in a double blind fashion; patients received either mint or placebo according to the code provided (in blocks of four at a ratio of 1:1) by the traditional pharmacy department of the Tehran University. The randomization code was not decoded until after the last patient completed all the experimental processes.

Physical and gynaecological examinations, ultrasound scan of uterus and ovaries, and measurement of laboratory parameters (pregnancy test, blood chemistry, and blood levels of FSH, LH, TSH, Prolactin, total Testosterone and free Testosterone) were performed at the beginning of study. Demographic and baseline data, medical history and any concomitant medications were also recorded. On the day of randomization, the patients were given a diary cards to complete during cycles 1 to 3 and were instructed on the correctly document drug intake (yes/no), vaginal bleeding and any complications of medications. Ultrasound scan of uterus and ovaries and measurement of plasma hormonal levels (LH, FSH, Total Testosterone and free Testosterone) were repeated at the end of the study. Recording of concomitant medication, compliance and any adverse events were performed at each visit. Pregnancy test was carried out with any delay menstruation at each stage of the study.

### Statistical analyses

The primary outcome measure was the occurrence (yes/no) of bleeding during the first treatment cycle. A bleeding episode was defined as bleeding if it occurred after consumption of medicinal herb.

Sample size was determined as follows. Based on Altman nomogram when the level of significance was α = 0.05 and the power to 80% and SD = 0.7, the number of patients required to complete each treatment arm was 30 to 35. Taking into account an expected dropout rate of 25%, the total number of patients required to investigate the primary efficacy variable was 84. In addition, in order to gain an insight into the regularity of bleeding in the cycles following one cycle bleeding, it was decided to recruit 120 patients.

The intention-to-treat (ITT) population used in the analysis of the primary endpoint comprised all randomized patients; those with missing diaries or missing data were considered as ‘failures’.

The secondary efficacy variable was the regularity of bleeding pattern during the three cycles of the study.

The ITT population for this analysis included all randomized patients who had documented data in the diary for three cycles (drug intake and bleeding data). The regularity of bleeding was scored by an index for each cycle as follows: an index of zero if no bleeding episode occurred, index of 1 if only one bleeding episode occurred, index of 2 if two times bleeding episode occurred and index of 3 if bleeding occurred in three consecutive cycles. The indices of all cycles were added together, resulting in a score ranging from 0 (absence of bleeding) to 3 (regular bleeding during all three cycles). These regularity scores were compared between groups using the Chi square test.

Further exploratory analyses were performed to compare the effects of mint and placebo on hormonal plasma levels, to determine the effects of stratification by “PCOS and FHA” and “secondary” amenorrhea and oligomenorrhea to evaluate any correlation between bleeding pattern and “PCOS and FHA” and”“secondary amenorrhea and oligomenorrhea”. The basis of these evaluations was the ITT population.

Comparisons between two groups were carried out by the student *t*-test and by the Chi square in the case of categorical data.

All tests in the exploratory analyses were carried out two-sided and a result with a *p* value of <0.05 was explained as significant. However due to multiple testing, the *p* values cannot be interpreted in a confirmatory sense as can the outcome for the primary efficacy variable.

All randomized women who received at least one dose of study drug were included in the safety analysis. Safety parameters included adverse events, physical examination findings, body weight, ultrasound scan of uterus and ovaries, concomitant medication, laboratory tests.

## Results

**Figure 2:**
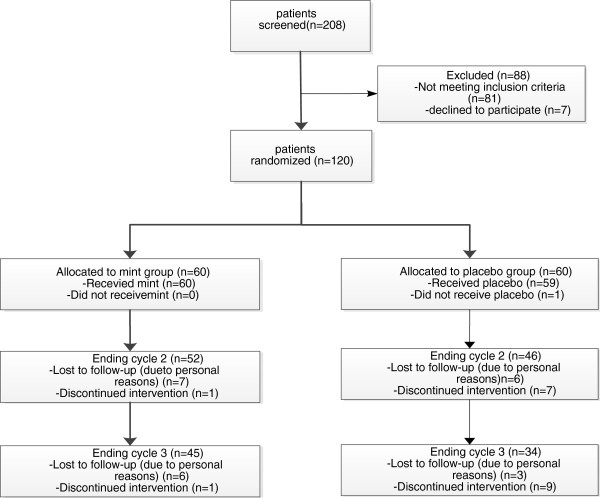
Distribution of patients.

Initially 208 patients were interviewed from which 120 patients were recruited and randomized in two groups of 60 patients. The distribution of patients during the study is shown in Figure 2.

The baseline and demographic characteristics of these patients are shown in Table 1; there were no relevant differences between the groups. 45 patients in the mint group and 34 patients in the placebo group completed the study. The subjects who had discontinued their therapy were higher in the placebo group (n = 26, 43.3%) than the mint group (n = 15; 25%; P < 0.05). The main reason of dropping out of the study was due to lack of effect, using hormonal drugs or a failure to follow up and personal reasons.

**Table 1:** Demographic and baseline characteristics

	**Mint**	**Placebo**	**P value**	**Statistical**
	**(n = 60)**	**(n = 60)**		**test**
Age (years), mean ± SD	24.63 ± 5.74	26.10 ± 5.71	0.1	*T* test
Weight (kg), mean ± SD	66.20 ± 16.70	66.97 ± 15.29	0.7	*T* test
Height (cm), mean ± SD	159.86 ± 6.3	161.15 ± 5.7	0.2	*T* test
BMI (kg/m^2^)	25.82 ± 5.93	25.80 ± 5.83	0.9	*T* test
PCOS, n (%)	44(73.3%)	49(81.7%)	0.1	Chi square
FHA, n (%)	16(26.7%)	11(18.3%)	0.1	Chi square
Secondary Amenorrhea, n (%)	45(75%)	40(66.7%)	0.2	Chi square
Oligomenorrhea, n (%)	15(25%)	20(33.3%)	0.2	Chi square

P value > 0.05.

*SD*, standard deviation; *BMI*, Body Mass Index; *PCOS*, Poly Cystic Ovarian Syndrome; *FHA*, Functional Hypothalamic Amenorrhea.

All 120 patients were included in the ITT analysis of the primary efficacy variable and in the safety analyses (Figure 1).

### Bleeding patterns

**Figure 3:**
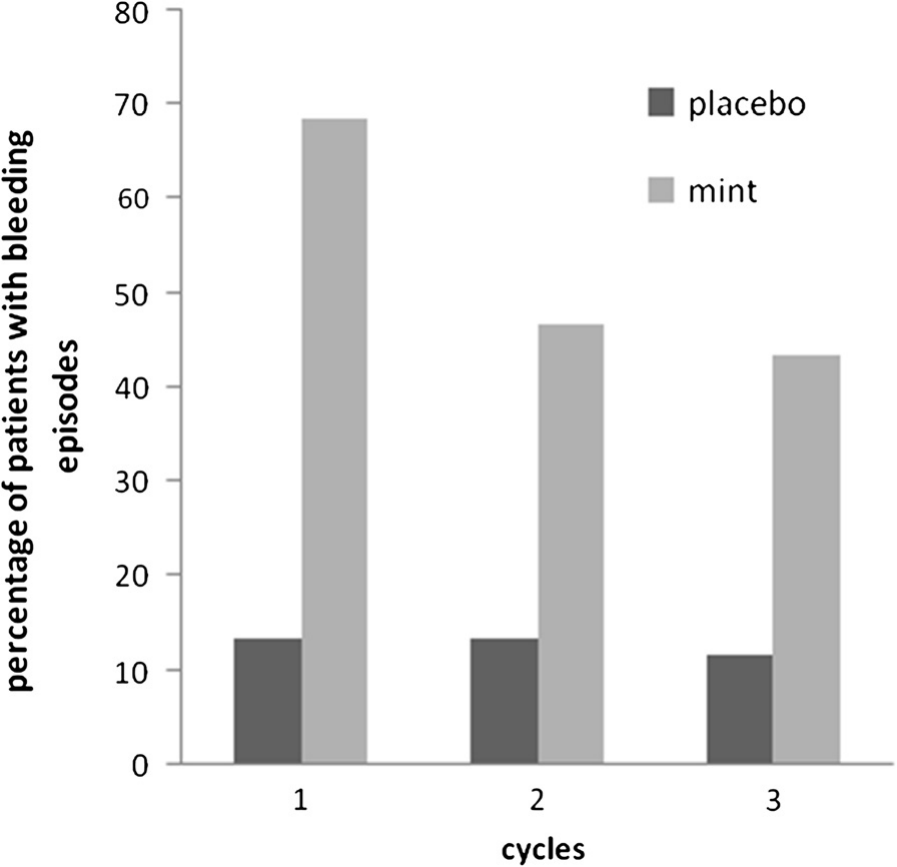
Percentage of patients with bleeding episodes per cycle during treatment with mint (n=60) or placebo (n=60) (Intention-to-treat (ITT) population).

The number of women with bleeding during the first cycle of treatment (the primary outcome) was higher in the mint group compared with the placebo group (68.3% vs. 13.6%; p < 0.001). Therefore the mint treatment was considered as more effective than placebo with regard to the primary efficacy variable (Figure 3).

During the three cycles of treatment, the percentage of patients who experienced bleeding episodes of any cycles was considerably higher with mint than with placebo (Figure 3). In both groups, on the other hand, the incidence of bleeding was dominantly high in the first cycle of treatment.

**Figure 4:**
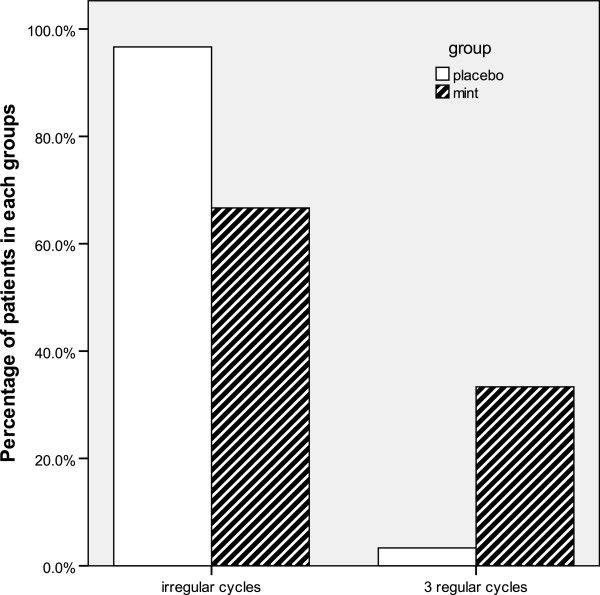
Percentage of patients with regular bleeding in all three cycles during treatment with mint (n=45) or placebo (n=34) (secondary efficacy variable).

The regularity of bleeding (regularity score) was significantly more consistent in patients treated with mint than in those given placebo (Table 2). In the mint group, 44 patients (73.3%) had regularity score of >0 compared with 18 patients (30%) in the placebo group (p < 0. 001) (Table 2). Regular bleeding in all three cycles (the secondary efficacy) was achieved in 33.3% (n = 20) of mint-treated patients but in placebo-treated patients only 3.3% (n = 2) experienced regular bleeding (p < 0. 001) (Figure 4).

**Table 2:** Regularity score by number of patients during treatment with mint or placebo (intention-to-treat population; n = 120)

**Menstruations Score**	**0**	**1**	**2**	**3**	**P value of Chi square**
Mint, n (%)	16 (26.7%)	13 (21.7%)	11 (18.3%)	20 (33.3%)	<0.001
Placebo, n (%)	42 (70%)	15 (25%)	1 (1.7%)	2 (3.3%)	

p < 0. 001.

Data are expressed as n (%), where n is number of patients.

Regularity scores of patients during treatment with mint or placebo (intention-to-treat population; n = 120) is shown in Table 2.

### Hormonal parameters

At the end of the treatment, there was a significant decrease in luteinizing hormone (LH) in mint group compared to placebo group (p < 0.002).

No changes of total testosterone, free testosterone and follicle-stimulating hormone (FSH) levels in the mint-treated group nor any changes of hormonal parameters in placebo group was recorded (Table 3).

**Table 3:** Pre- and post-treatment TT, FT, LH, FSH

**variables**	**Group**	**Pre-treatment**	**P value of*T*test**	**Post-treatment**	**P value of*T*test**
TT (ng/mL)	Mint	0.7 ± 0.28	0.2	0.51 ± 0.29	0.2
	Placebo	2.01 ± 8.8		0.65 ± 0.59	
FT (ng/mL)	Mint	1.68 ± 1.12	0.8	1.35 ± 0.94	0.3
	Placebo	1.65 ± 1.15		1.62 ± 1.28	
LH (mlU/mL)	Mint	8.9 ± 7.4	0.2	5.6 ± 4.6	0.002
	Placebo	10.5 ± 6.4		10.9 ± 5.8	
FSH (mlU/mL)	Mint	6.65 ± 11.8	0.4	4.62 ± 1.68	0.9
	Placebo	5.3 ± 2.03		4.6 ± 1.1	

*TT*, total testosterone; *FT*, free testosterone; *LH*, Luteinizing hormone; *FSH*, follicle-stimulating hormone.

### Subgroups analyses

Comparing the subgroups of patients with PCOS (n = 93) and FHA (n = 27) with respect to demographic parameters revealed significantly (p < 0.001) more patients in the PCOS subgroup than in the FHA subgroup (Table 1). The subgroups of patients with PCOS and FHA, the incidence of bleeding during the first cycle (37.6% vs. 51.9%; p = 0.378) and regular bleeding in all three cycles (15.1% vs. 29.6%; p = 0.096) was not significantly different.

The subgroups of patients with secondary amenorrhea (n = 85) and oligomenorrhea (n = 35) showed significantly (p < 0.001) more in the secondary amenorrhea subgroup than in the oligomenorrhea subgroup (Table 1). Among subgroups of secondary amenorrhea and oligomenorrhea, the incidence of bleeding during the first cycle (40% vs. 42.9%; respectively p = 0.27) and regular bleeding in all three cycles (16.5% vs. 22.9%; respectively p = 0.442) was not significantly different.

There were no other significant differences between the four subgroups with regard to demographic and other baseline characteristics.

### Safety and tolerability

Mint syrup was well tolerated by the patients. There were no serious adverse reactions in the mint group. Complication were classified as pleasant or unpleasant and were reported in 19 patients (31.7%) treated with mint syrup. The most common unpleasant reports were spotting (n = 7), severe bleeding (n = 2), constipation (n = 1) and stomachache (n = 1) were seen in the mint-treated group.

The most common pleasant effects were reduced gastrointestinal complaints such as reduced bloating (n = 2), abdominal cramps (n = 1), nausea (n = 1), stomachache (n = 1). Another good side effect was decreasing dysmenorrhea (n = 2).

## Discussion

The present study is the first comprehensive assessment of menstruation upon *Mentha longifolia L.* ingestion in the context of a large randomized placebo-controlled trial in women with secondary amenorrhea and oligomenorrhea.

The results from this study showed that sequential mint intake has statistically significant advantages over placebo with regard to the induction of uterus bleeding and the regularity of the menstruation.

The percentage of patients experiencing uterus bleeding during the first cycle was 68.3% in the mint group and 13.6% in the placebo group. The percentage of patients with bleeding was highest during the first cycle and tended to reduce thereafter in both groups.

The regularity of bleeding throughout the study was markedly improved in the mint group. Regular bleeding during three cycles was achieved in one third of the patients treated with mint compared to those given placebo (33.3% vs 3.3%).

To our knowledge no study has been performed yet to evaluate the effects of this herbal drug on uterus bleeding. Due to the lack of similar studies with herbal remedies, the results of other studies are reviewed.

In a study by Panay et al. on 104 patient to assess the efficacy of dydrogesterone in inducing regular withdrawal bleeding, the number of women with withdrawal bleeding during the first cycle was twice as high in the dydrogesterone group as in the placebo group (65.4% vs. 30.8%; p < 0.0004). Superiority of dydrogesterone was also observed for regularity of bleeding over the six cycles (p < 0.0001) [2].

In Dessole et Al. studied, they used a low dose (75 IU/day for 5 days) of purified FSH (Metrodin) in 10 oligomenorrheic patients (aged 18–30) for 25 cycles. The onset of menstruation occurred in 7 patients (70%) and in 19 treatment cycles (76%), the ovulation was verified in 5 of these patients (50%) for 13 cycles (52%). One patient had spotting after the treatment, two patients did not have any response [20].

This study revealed that the consumption of *Mentha longifolia L.* syrup will decrease LH levels. In a study by Shariati M et al., the effect of Lamiacea family on FSH was investigated. Assessment of the effect of Mentha pulegium leaves on gonadotropin tests in male rat showed that the hydro-alcoholic extract of Mentha pulegium leaves causes a significant decrease in FSH and LH levels [21].

In contrast, another experimental study by Akdogan M et al. on effects of Mentha piperita and Mentha spicata on plasma androgen and FSH and LH levels, it was reported that the consumption of M. piperita and M. spicata can decreased plasma testosterone and increase the plasma LH and FSH levels in rats [22]. Because antiandrogenic effects of spearmint and peppermint were found in rats, they observed the effect of this herbal tea on the androgen levels in hirsute women so Akdogan M et al., choose twenty-one female hirsute patients, were took a cup of herbal tea which was steeped with M. spicata for 5 days twice a day in the follicular phase of their menstrual cycles. After treatment with spearmint teas, there was a significant decrease in free testosterone and increase in LH and FSH levels. There were no significant decreases in total testosterone [23].

The dropout rate in the placebo group (n = 26, 43/3%) were higher than the treatment group (n = 15; 25%; P < 0.05). Total dropout is more than other studies.

We need to notice some limitations that we have face in present study. These limitations are generally common in studies involving human subjects. First, the dropout rate in this study was more than usual, due to the long duration of treatment and interference with personal and cultural issues. Second, the Iranian traditional medicine have one major variable which be present in human including temperament (mezaj) and racial/ethnic, sex, age, area, season, job and etc. [14,24]. These variations can effect in bioavailability differences in human. Indeed in this study our subjects were unable to assess individual differences in patients according to traditional medicine.

## Conclusion

The results of this study showed that *Mentha longifolia L.* syrup is significantly effective over placebo in inducing bleeding in women with secondary amenorrhea and that the pattern of bleeding is significantly more regular with *Mentha longifolia L.*, while side effects are least.

Due to the beneficial effects of *Mentha longifolia L.*, besides its safety, availability and low cost; a future therapeutic role in women with amenorrhea and oligomenorrhea is expected.

However, further large randomized studies are needed to determine appropriate dosages and duration of treatment and the reliability of *Mentha longifolia L.* as a good option for cessation of menstruation.

## Abbreviations

HRT: Hormone replacement therapy; ITM: Iranian traditional medicine; SA: Secondary amenorrhea; FHA: Functional hypothalamic amenorrhea; PCOS: Poly cystic ovarian Syndrome; FSH: Follicle-Stimulating Hormone; LH: Luteinizing hormone; ITT: Intention-to-treat; BMI: Body Mass Index; TT: Total testosterone; FT: Free testosterone.

## Competing interests

The authors do not have any financial/commercial competing interest in the study presented here.

## Authors’ contributions

RM has made substantial contribution in designing, acquisition of data, and drafting the manuscript and has given the final approval of the version to be published. NZ the supervisor of conduction of the study, participated involved in design, interpretation of data, and revising, have given final approval of the version to be publish. SB study researcher, involved in design, and revising. FHD analyzed and interpreted the data. MN co- study designer, and revising. MK participated involved in revising. GHA participated in the identification of the plants, plant extraction and made substantial contributions in the study. AG participated in the study design and conceptions. MT participated involved have made substantial contributions to conception, and revising. AA participated in doing laboratory tests and revising. MAHT involved in the study design and conceptions. All authors read and approved the final manuscript.
